# Evaluating postgraduate family medicine supervisor feedback in registrars’ learning portfolios

**DOI:** 10.4102/phcfm.v14i1.3744

**Published:** 2022-12-20

**Authors:** Neetha J. Erumeda, Ann Z. George, Louis S. Jenkins

**Affiliations:** 1 Department of Family Medicine and Primary Care, Faculty of Health Sciences, University of the Witwatersrand, Johannesburg, South Africa; 2 Gauteng Department of Health, Ekurhuleni District Health Services, Germiston, South Africa; 3 Centre of Health Science Education, Faculty of Health Sciences, University of the Witwatersrand, Johannesburg, South Africa; 4 Division of Family Medicine and Primary Care, Department of Family and Emergency Medicine, Faculty of Health Sciences, Stellenbosch University, Cape Town, South Africa; 5 George Hospital, Western Cape Department of Health, George, South Africa; 6 Primary Health Care Directorate, Department of Family, Community and Emergency Care, Faculty of Health Sciences, University of Cape Town, Cape Town, South Africa

**Keywords:** decentralised training, family physician, feedback, individualised learning plan, learning portfolio, postgraduate supervision, mini-Clinical Evaluation Exercise

## Abstract

**Background:**

Postgraduate supervision forms a vital component of decentralised family medicine training. While the components of effective supervisory feedback have been explored in high-income countries, how this construct is delivered in resource-constrained low- to middle-income countries has not been investigated adequately.

**Aim:**

This article evaluated supervisory feedback in family medicine registrars’ learning portfolios (LPs) as captured in their learning plans and mini-Clinical Evaluation Exercise (mini-CEX) forms and whether the training district or the year of training affected the nature of the feedback.

**Setting:**

Registrars’ LPs from 2020 across five decentralised sites affiliated with the University of the Witwatersrand in South Africa were analysed.

**Methods:**

Two modified tools were used to evaluate the quantity of the written feedback in 38 learning plans and 57 mini-CEX forms. Descriptive statistics, Fisher’s exact and Wilcoxon rank-sum tests were used for analysis. Content analysis was used to derive counts of areas of feedback.

**Results:**

Most learning plans (61.2%) did not refer to registrars’ clinical knowledge or offer an improvement strategy (86.1%). The ‘extent of supervisors’ feedback’ was rated as ‘poor’ (63.2%), with only 14.0% rated as ‘good.’ The ‘some’ and ‘no’ feedback categories in the mini-CEX competencies (*p* < 0.001 to *p* = 0.014) and the ‘extent of supervisors’ feedback’ (*p <* 0.001) were significantly associated with training district. Feedback focused less on clinical reasoning and negotiation skills.

**Conclusion:**

Supervisors should provide specific and constructive narrative feedback and an action plan to improve registrars’ future performance.

**Contribution:**

Supervisory feedback in postgraduate family medicine training needs overall improvement to develop skilled family physicians.

## Introduction

Feedback is a vital aspect of supervision in clinical settings.^1,2^ Feedback is defined as ‘a way the trainee comes to know about the gaps in their current level of knowledge and the desired goal’.^3^ For maximum effectiveness, feedback should be provided timeously, in small, manageable quantities, relate to observable behaviours^4^ and should be tailored to the trainee’s learning needs and non-judgemental.^5,6^ Feedback must also be clearly articulated based on trainee’s observed performance,^7^ promote self-assessment,^8^ be context-specific, enable interactions between the trainee and supervisor^3^ and focus on adequate performance and areas for improvement.^9,10^ Supervisors should assist trainees in developing written action plans specifying the supervisor’s role in supporting trainees to achieve the goals outlined in the learning plans.^9,10^ More recent definitions regard feedback as a coaching activity where supervisors facilitate trainees’ self-reflection to improve performance.^10,11^ However, the acceptability of feedback by trainees still depends on who provides it, whether it is constructive, the trainee’s assessment literacy and the nature of supervisor-trainee relationships.^5,11^

### The role of feedback in postgraduate family medicine training

Postgraduate family medicine decentralised clinical training of FPs involves educational and clinical supervision, each of which can be demonstrated in trainees’ learning portfolios (LPs). Educational supervision includes identifying trainees’ learning objectives and providing feedback on their personal development plans and progress in their LPs.^12,13^ Clinical supervision involves giving feedback on trainees’ relationships with staff and patients, their management of clinical conditions and their professionalism.^14^ The LPs provide evidence of trainees’ observed performance by clinical supervisors using workplace-based assessments (WPBAs),^15^ using tools such as the mini-Clinical Evaluation Exercise (mini-CEX) form.^16^

### Educational supervision in learning plans

Individualised learning plans in LPs are recognised as essential components of self-regulated lifelong learning,^17^ which refers to trainees’ ability to modify their thinking behaviour and level of motivation.^18^ Learning plans are essential tools for postgraduate trainees, assisting them with understanding their strengths, professional goals and the family medicine speciality requirements needed to make individual adjustments and identify resources to progress.^19^ The educational supervisor’s role in the learning plans is to orientate trainees to the learning environment, help them set goals and objectives and plan feedback sessions.^6^

Learning plans can also promote self-directed learning, which differs from self-regulated learning. In self-directed learning, trainees develop their learning goals, identify activities and resources to achieve them and get external feedback to modify learning. By comparison, in self-regulated learning, the trainers set the goals, and trainees regulate their learning, influenced by their cognitive domain, which primarily happens in an academic environment.^20^ However, there is a lack of literature on self-directed learning,^20^ especially on the potential for learning plans to promote self-directed learning based on the quality of the feedback provided. The self-directed learning goals trainees develop in these plans must be meaningful, specific, measurable, accountable, realistic and include specified timelines.^21^ While internal accountability facilitates self-reflection, trainees’ external accountability requires regular supervisor feedback.^21^

### Clinical supervision in the mini-Clinical Evaluation Exercise

The mini-CEX tool^16^ is widely used in HICs to assess clinical competencies.^22,23,24^ Although this tool was modified to accommodate several scoring systems, clinical disciplines and settings,^5,22^ more contextualised studies are needed to understand its effectiveness in resource-limited settings. One of the primary purposes of the mini-CEX form is to provide structured feedback on observed performance.^5,22^ The educational impact of this tool^24^ is enhanced when combined with well-written feedback^25^ and negatively impacted by inadequate feedback.^5,23^ Supervisors often provide inadequate feedback by, for example, not appreciating or being aware of the role of feedback as an educational tool, not being skilled enough to provide helpful feedback, and tending to give high numerical scores (superior or satisfactory) on each mini-CEX competency,^25^ without explaining why this score is justified. Trainees value narrative feedback more than numerical scores-narrative feedback helps them to distinguish between performance deemed ‘good’ and ‘not so good’, promotes reflection on their performance,^26^ and develop an action plan.^27^

In sub-Saharan Africa, clinical supervisors play a crucial role in postgraduate training in the workplace.^28^ Clinical supervisors are expected to provide constructive, good-quality verbal and written feedback during WPBAs. An evaluation of supervisors’ feedback in registrars’ LPs at a South African university showed that feedback was better documented in e-portfolios than in paper-based ones but was still not sufficiently specific.^29^ A satisfactory LP is a prerequisite for registrars (trainees) to sit the national exit examination to qualify as an FP.^30^ One of the challenges of tools such as learning plans or mini-CEX used in LPs is that they assess ‘hard’ skills (history-taking, physical examination and management) and ‘soft’ skills (problem-solving, communication, collaboration and professionalism).^20^ While some received training in providing feedback in South Africa,^31^ many FPs may not know what constitutes adequate feedback, which is more complex for ‘soft’ skills.

Evaluating feedback in learning plans or the mini-CEX tool is still in its infancy in various high and low-middle income countries in clinical settings, including sub-Saharan Africa. It has been identified as an area for further research.^20,27^ This article evaluated both the quantity and quality of supervisory feedback and the scores given in registrars’ LPs as captured in their learning plans and mini-CEX forms. The article also reports whether the training district or year of training affected the nature of the feedback across the decentralised clinical training sites. The results could help develop recommendations for improving educational and clinical supervision feedback in similar settings nationally and across sub-Saharan Africa.

## Research method and design

### Study design and setting

This study is part of a broader cross-sectional, convergent mixed-methods study that evaluated postgraduate family medicine training at five decentralised training sites affiliated with the University of the Witwatersrand in South Africa, using the logic model. The logic model is causal, based on the reductionist theory that links the inputs, processes, outputs and outcomes.^32^ A previous publication from the broader study reported evaluating postgraduate supervision as a process in the logic model.^33^ This article reports evaluating supervisors’ feedback as a proxy measure for effective supervision as an output in the logic model. In the setting for this study, registrar training is primarily based in primary or community healthcare clinics or a district hospital. During clinical rotations, registrars join various disciplines for 3 months or less in regional hospitals to gain specific knowledge and skills. Family medicine registrars are primarily supervised by FPs but may be supervised by non-family physician (non-FP) supervisors during clinical rotations. The non-FP supervisors could be specialists, registrars in other disciplines, or medical officers. Medical officers are general practitioners working at hospitals or clinics without any specialist registration.

Family physician training for their supervisory role varies. Some FP supervisors had attended ‘train the trainer’ courses conducted by the South African Academy of FPs. Others had opportunities to attend the courses organised by Wits University but were not trained formally in medical education. The background of the non-FP supervisors in medical education training was not well understood at the time of this study. Family physicians act as educational supervisors and mentors, assess registrars’ learning plans and provide feedback during clinical rotations. In addition, as part of WPBAs, FP supervisors complete mini-CEX forms during directly observed consultations by registrars with patients in clinical settings. Supervisors’ written feedback in the learning plans and mini-CEX forms provides evidence for the quality of clinical and educational supervision.

This study evaluated the written feedback in registrars’ LPs from the five decentralised training sites, four of which are in Gauteng province and the fifth in the Northwest province.

### Study population and sampling

All LPs submitted in 2020 by the 20 registrars across all 3 years of training were eligible for evaluation, constituting a purposive sample.^34^ Thirty-six learning plans and 57 mini-CEX forms were evaluated from 19 eligible LPs. One portfolio was excluded as there was no consent. The learning plans and mini-CEX forms represented all 5 training districts (D1–D5) and 3 years of training (Y1–Y3).

### Data collection

The authors evaluated the learning plans and mini-CEX forms between October and December 2020.

#### Learning plans

At the beginning of a clinical rotation, the registrar develops goals in their learning plan. The supervisor then provides initial feedback on these goals. At the end of the rotation, the supervisor assigns a global score for the registrar’s knowledge, skills, professional values, and attitudes and provides final written feedback. A validated tool developed at a South African university^29^ was modified to assess supervisors’ final feedback in the learning plans. The tool was adapted in consultation with one of the co-authors, who developed the validated tool: variables were added to make it more comprehensive on each of the feedback categories extracted from the data, thus increasing the construct validity of the tool.

The modified tool consists of seven categories:

- feedback on registrars’ professional behaviour
- feedback on registrars’ knowledge and skills
- the valence of feedback
- the presence of an improvement strategy
- a rating of the extent of supervisors’ feedback
- the signatory (FP or other clinical supervisors) on the initial feedback
- the signatory (FP or other clinical supervisors) on the final feedback.

The written feedback given by supervisors in the general feedback section was captured in an MS Excel spreadsheet to analyse the quality. The spreadsheet also recorded the training district, year of training and global numerical scores from each learning plan were also recorded in Microsoft Excel. The rating of the extent of feedback was classified as ‘poor’, ‘average’, or ‘good’ by the researchers, depending on the feedback aspects provided. The feedback was rated ‘poor’ if (1) feedback was absent or irrelevant (2) specific feedback was not provided for the following categories in the tool: professional behaviour, knowledge and skills, valence of feedback and improvement strategy present, (3) forms were not signed and completed by the supervisor. A rating of ‘average’ was assigned when specific feedback was provided on one or two of the categories (registrar professional behaviour, knowledge and skills, valence of feedback provided or improvement strategy) and forms were signed and completed. The rating was ‘good’ when feedback was provided for three or more categories (registrar professional behaviour, knowledge and skills, valence of feedback and improvement strategy) and the form was signed and completed.

#### Mini-Clinical Evaluation Exercise forms

The mini-CEX form requires supervisors to allocate individual scores for the competencies and provide a total score. The form includes sections for the supervisor’s feedback and supervisor and registrar signatures.

We developed a feedback assessment tool to evaluate written feedback in the mini-CEX forms, whether explicitly related to the seven competencies or appearing in the general feedback section. The tool assessed the extent and adequacy of the supervisors’ feedback, the training district, the year of training, and whether both FPs and registrars signed the form. The ‘extent of feedback’ rating was classified as ‘poor’, ‘average’, or ‘good’ depending on the areas of mini-CEX competencies addressed, whether supervisors had completed the general feedback section and whether the supervisor and registrar signed the forms. The scores for each of the seven competencies and total scores given in mini-CEX forms were recorded in Microsoft Excel. The narrative feedback provided in the general feedback section was captured in a separate MS Excel spreadsheet to analyse the quality.

### Data analysis

The data were analysed for descriptive statistics using STATISTICA 13.5.0.17 and for associations using STATA 14.2. For the inferential statistics, the feedback categories were collapsed into ‘some feedback’ and ‘no feedback’ in both learning plans and mini-CEX forms. For both tools, the absence of feedback was categorised as ‘no feedback’, and all other feedback categories were grouped as ‘some feedback’. Similarly, if the rating of ‘extent of supervisor’s feedback’ was ‘poor’, this was categorised as ‘no feedback’, and the ‘average’ and ‘good’ categories were grouped into a single category labelled ‘some feedback’. All associations between ‘no feedback’ and ‘some feedback’ categories and the training district or the year of training were determined using Fisher’s exact tests (*p* < 0.05). Fisher’s exact tests were used rather than chi-squared tests because of the small sample size.^35^ Where there was a statistically significant association, Fisher’s exact tests were used to test each training district and year with the ‘some feedback’ and ‘no feedback’ categories to determine the strength of association.

Kruskal–Wallis tests were used to compare the global and total scores in the learning plans and mini-CEX forms across the training district and year of training. Two-sample Wilcoxon rank-sum tests were used to test the strength of association. Bonferroni correction was applied for 2X2 training district and year of training comparison with feedback categories and scores and significance for the *p*-value was set at < 0.005 and < 0.017, respectively.

The feedback was first characterised according to length, that is, the number of words, overall quality and specificity to evaluate the quality of written narrative feedback in both forms. The characteristics assessed for the overall feedback quality in this study were extracted from validated tools used in previous studies. The following feedback quality characteristics were adapted from De Swardt et al. (2019) the number of words in the feedback, whether proper sentences were used, strengths and weaknesses highlighted and areas of improvement specified.^29^ The following were derived from Pilgrim et al.: whether an action plan was provided and whether the feedback was specific.^36^ For example, if the feedback simply read ‘satisfactory’ or ‘good’, it was coded under non-specific feedback. The second level of analysis involved content analysis^37^ to derive counts for specific feedback areas. For example, the feedback ‘the joint examination was performed well’ was coded as ‘used proper sentences’ and ‘provided strengths’ under the overall quality. This feedback example was coded in the category ‘physical examination’ during the content analysis. The main author and co-authors initially separately, coded the data set, then compared their coding systems. Any differences were discussed until agreement on the naming of codes and categories and the categorisation of the codes was reached. The coding process was iterative, involving several cycles of discussions until the data analysis was completed. The iterative process improved the interrater reliability of the findings.^38^

### Ethical considerations

Ethical approval for the broader project was obtained from the Human Research Ethics Committee (Medical) of the University of the Witwatersrand (certificate number: M191140). Permission to conduct the research was obtained from the University Registrar and the Head of the Department of Family Medicine. Informed written consent to access the LPs was obtained from registrars and supervisors.

## Results

When the feedback in the 36 learning plans was analysed, most (61.2%) did not refer to registrars’ clinical knowledge or skills, and 86.1% did not offer an improvement strategy. The ‘extent of supervisors’ feedback’ was mainly rated as ‘poor’ (47.2%) or ‘average’ (47.2%); only a few (5.6%) were ‘good’. In one-third of the forms, the final feedback was provided by non-FP supervisors from other clinical departments (Table 1). The data were not uniformly distributed, so the global score median of 8.0 (7.7–9.0) out of 10.0 in the learning plans was calculated.

**TABLE 1:** Learning plan feedback characteristics (*N* = 38).

Variable	*n*	%
**Training district**		
District 1	10	27.7
District 2	6	16.7
District 3	6	16.7
District 4	8	22.2
District 5	6	16.7
**Year of training**		
Year 1	10	27.8
Year 2	16	44.4
Year 3	10	27.8
**Reference to registrar’s professional behaviour**		
No feedback	15	41.7
General feedback	8	22.2
Specific feedback	13	36.1
**Reference to registrars’ clinical knowledge or skills**		
No feedback	22	61.2
General feedback	7	19.4
Specific feedback	7	19.4
**Statement indicating valence of feedback**		
No feedback and/or unclear	15	41.7
Mostly positive or negative	20	55.6
Constructive feedback (positive balanced with negative)	1	2.7
**Comments offering an improvement strategy**		
No feedback	31	86.1
General feedback	3	8.3
Specific feedback	2	5.6
National unit standards	0	0.0
**Initial feedback given and completed by**		
Family physician supervisor	24	66.7
Academic coordinator	8	22.2
Other specialists	1	2.8
Medical officer	0	0.0
Not signed	3	8.3
**Final feedback given and completed by**		
Family physician supervisor	16	44.4
Academic coordinator	8	22.2
Other specialists	8	22.2
Medical officer	4	11.2
**Rating of the extent of supervisors’ feedback**		
Poor (No feedback; irrelevant feedback; no specific feedback on at least one aspect; not signed and completed)	17	47.2
Average (average or good feedback on one to two aspects; signed and completed)	17	47.2
Good (gives feedback on three or more aspects; signed and completed)	2	5.6

In 17 of the 19 LPs, the mini-CEX forms were completed by more than one FP from the same district, while the same FP completed the remaining two. For the ‘extent of supervisors’ feedback’, 63.2% were classified as ‘poor’ and only 14.0% as ‘good’ (Table 2). The median of the total scores in the mini-CEX forms was 6.8 (range: 6.1–7.3). Most of the mini-CEX competency scores given by FP supervisors ranged between 5–7 or 8–10, with very few scores of 1–4 (Table 3).

**TABLE 2:** Mini-Clinical Evaluation Exercise feedback characteristics (*N* = 57).

Variable	*n*	%
**Training district**		
District 1	15	26.3
District 2	12	21.1
District 3	9	15.7
District 4	12	21.1
District 5	9	15.8
**Year of training**		
Year 1	18	31.6
Year 2	21	36.8
Year 3	18	31.6
**General Feedback**		
No feedback (1)	10	17.5
Feedback on areas performed well (2)	3	5.3
Feedback on areas not performed well (3)	4	7.0
Areas performed well and not well (4)	5	8.8
Offer strategies for improvement (5)	11	19.3
General or reference to national unit standards (6)	2	3.5
Combinations of 2–4 and 5	15	26.3
Combinations of 2–4 and 6	7	12.3
**Rating of the extent of supervisors’ feedback**		
Poor (feedback on ≤ 2 areas of competencies; no general feedback)	36	63.2
Average (feedback on 3–4 areas of competencies; general feedback completed)	13	22.8
Good (feedback on five or more areas of competencies; general feedback completed)	8	14.0
**Signed by registrar and supervisor**		
Completed	29	50.9
Not completed	28	49.1

**TABLE 3:** Feedback characteristics and scores on mini-Clinical Evaluation Exercise competencies (*N* = 57).

Mini-CEX competencies	Establish relationship		Gathering information		Physical examination		Clinical judgement		Explaining and planning		Shows organised approach		Overall competence	
	*n*	%	*n*	%	*n*	%	*n*	%	*n*	%	*n*	%	*n*	%
**Feedback categories**														
No feedback	40	70.2	29	50.9	35	61.4	39	68.4	40	70.2	44	77.2	48	84.2
Feedback on areas performed well	10	17.5	12	21.1	8	14.0	10	17.6	10	17.5	4	7.0	3	5.7
Feedback on areas not performed well	2	3.5	11	19.3	12	21.1	7	12.3	4	7.0	7	12.3	4	7.0
Areas performed well and not performed well	1	1.8	4	7.0	2	3.5	1	1.8	1	1.8	0	0.0	0	0.0
Feedback general or inadequate	4	7.0	1	1.8	0	0.0	0	0.0	2	3.5	2	3.5	2	3.5
**Score categories**														
Score (1–4)	0	0.0	1	1.8	4	7.1	0	0.0	0	0.0	1	1.8	0	0.0
Score (5–7)	34	59.7	44	77.2	43	75.4	43	75.4	43	75.4	43	75.4	46	80.7
Score (8–10)	22	38.6	12	21.1	9	15.8	14	24.6	14	24.6	13	22.8	11	19.3
No score	1	1.8	0	0.0	1	1.8	0	0.0	0	0.0	0	0.0	0	0.0

Mini-CEX, mini-Clinical Evaluation Exercise.

Further analysis showed a statistically significant association of the ‘some feedback’ and ‘no feedback’ categories with all Mini-CEX competencies (*p* < 0.001 to *p* = 0.014), the rating of ‘extent of supervisors’ feedback’ (*p* < 0.001) and the ‘signed by registrar and supervisor’ (*p* < 0.001) categories with the training district (Table 4). However, there was no association with the year of training. There was no significant association of feedback categories in the learning plans with either the training district or year of training.

**TABLE 4:** Association of mini-Clinical Evaluation Exercise feedback categories with training district and year of training.

Mini-CEX competencies	Training district										*p* †	Year of training						*p* †
	D1		D2		D3		D4		D5			Y2		Y3				
	*n*	%	*n*	%	*n*	%	*n*	%	*n*	%		*n*	%	*n*	%	*n*	%	
**Establish relationship**											< 0.001							0.128
Some feedback	1	4.5	4	3.6	8	2.7	3	3.6	1	2.7		2	5.4	8	6.3	7	5.4	
No feedback	14	10.5	8	8.4	1	6.3	9	8.4	8	6.3		16	12.6	13	14.7	11	12.6	
**Gathering information**											0.009							0.241
Some feedback	3	7.4	8	5.9	8	4.4	6	5.9	3	4.4		6	8.8	13	10.3	9	8.8	
No feedback	12	7.6	4	6.1	1	4.6	6	6.1	6	4.6		12	9.2	8	10.7	9	9.2	
**Physical examination**											< 0.001							0.466
Some feedback	3	5.8	9	4.6	7	3.5	2	4.6	1	3.5		7	6.9	10	8.1	5	6.9	
No feedback	12	9.2	3	7.4	2	5.5	10	7.4	8	5.5		11	11.1	11	12.9	13	11.1	
**Clinical judgement**											0.014							0.287
Some feedback	3	4.7	4	3.8	7	2.8	1	3.8	3	2.8		3	5.7	8	6.6	7	5.7	
No feedback	12	10.3	8	8.2	2	6.2	11	8.2	6	6.2		15	12.3	13	14.4	11	12.3	
**Explaining and planning**											< 0.001							0.765
Some feedback	0	4.5	6	3.6	8	2.7	2	3.6	1	2.7		6	5.4	7	6.3	4	5.4	
No feedback	15	10.5	6	8.4	1	6.3	10	8.4	8	6.3		12	12.6	14	14.7	14	12.6	
**Show organised approach**											<0.001							0.794
Some feedback	0	3.4	2	2.7	8	2.1	3	2.7	0	2.1		3	4.1	5	4.8	5	4.1	
No feedback	15	11.6	10	9.3	1	6.9	9	9.3	9	6.9		15	13.9	16	16.2	13	13.9	
**Overall competence**											< 0.001							0.902
Some feedback	0	2.4	3	1.9	6	1.4	0	1.9	0	1.4		3	2.8	4	3.3	2	2.8	
No feedback	15	12.6	9	10.1	3	7.6	12	10.1	9	7.6		15	15.2	17	17.7	16	15.2	
**Rating of the extent of supervisors’ feedback**											< 0.001							0.932
Some feedback	2	5.5	9	4.4	8	3.3	2	4.4	0	3.3		7	6.6	8	7.7	6	6.6	
No feedback	13	9.5	3	7.6	1	5.7	10	7.6	9	5.7		11	11.4	13	13.3	12	11.4	
**Signed by registrar and supervisor**											< 0.001							0.944
Completed	1	6.8	6	5.8	4	4.3	7	5.8	9	4.3		9	8.2	10	10.1	8	8.7	
Not completed	13	7.3	6	6.2	5	4.7	5	6.2	0	4.7		8	8.8	11	10.9	10	9.3	

Mini-CEX, mini-Clinical Evaluation Exercise; D, district; Y, year.

† , Fisher’s exact test, *p*< 0.05.

When the strength of association of D1–D5 with the feedback categories was tested further, D3 feedback rated higher when compared with other districts in all mini-CEX competencies with a statistical significance (*p* < 0.001 to *p* = 0.003). The ‘extent of supervisors’ feedback was also rated higher in D2 and D3 than D1, D4 and D5, which was significantly different (*p* < 0.001 to *p* = 0.002). D1 had significantly more signed and completed forms than D5 (*p* < 0.001).

Comparing the global scores in learning plans and total scores in mini-CEX forms with the year of training revealed significant differences in the median values of the global score (*p* = 0.012) and the total score (*p* = 0.049). On further analysis with two-sample Wilcoxon rank-sum tests, the differences were between Y1 and Y3 in both global score (*p* = 0.003) and the total score (*p* = 0.016). There were no significant differences between Y1 and Y2, or Y2 and Y3, across both forms. There were no significant differences in the median global and total scores across D1–D5.

**FIGURE 1:**
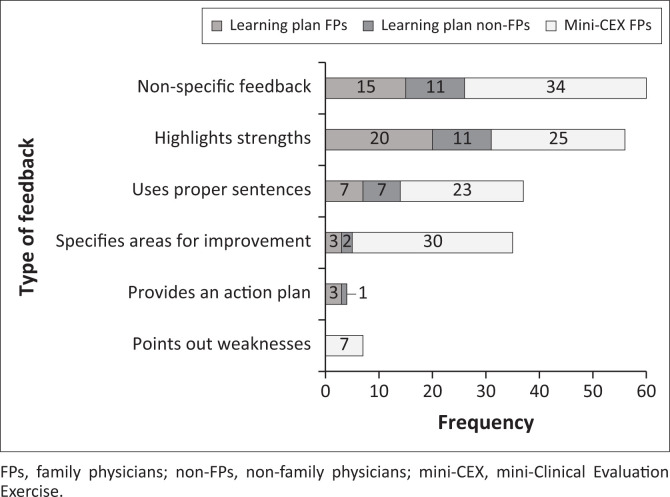
Overall quality of feedback in learning plans and mini-Clinical Evaluation Exercise forms.

**FIGURE 2:**
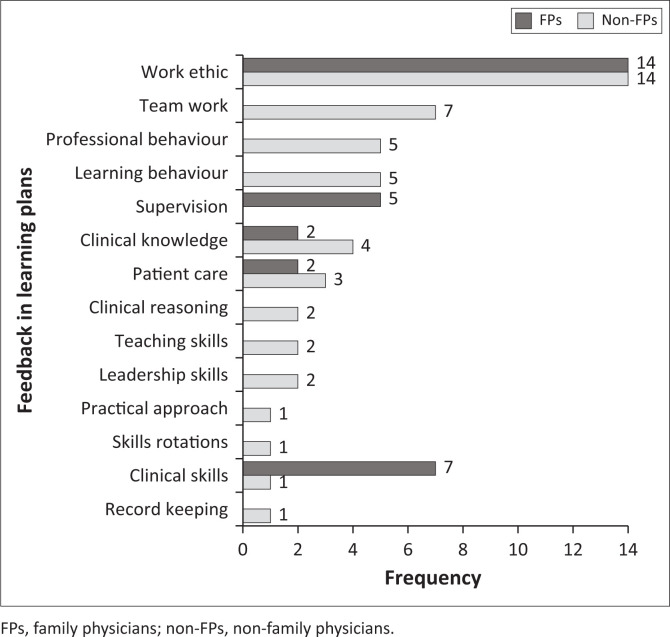
Areas of feedback in learning plans by family physicians and non-family physicians.

The FP supervisors had provided the feedback in 22/36 learning plans, non-FP supervisors in 12/36 and 2/36 had no feedback. Non-FP supervisors provided more extended feedback (average of −25.8 words) than FP supervisors (average of −13.7 words). The feedback focused mainly on ‘highlighting strengths’ or was ‘non-specific’, with less emphasis on ‘specific areas for improvement’ and ‘providing action plans’ (Figure 1). The FP feedback focused only on registrars’ ‘work ethic’ and ‘clinical skills’. In contrast, non-FP feedback was more comprehensive because it referred to various areas, including ‘professional behaviour’ and ‘teamwork’ (Figure 2).

**FIGURE 3:**
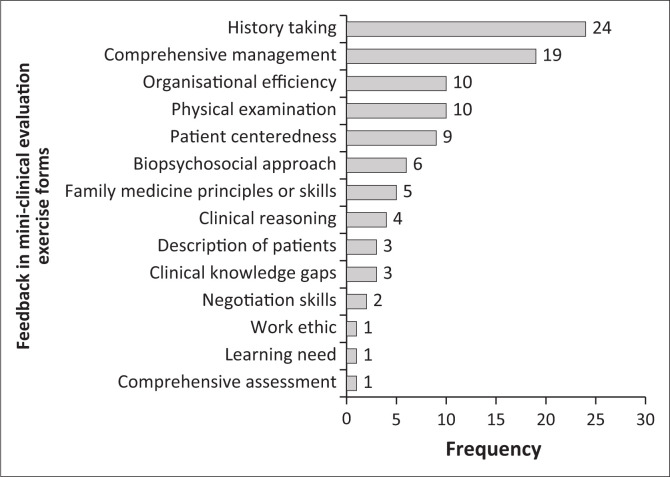
Areas of feedback in mini-clinical Evaluation Exercise forms by family physicians.

The FPs mini-CEX feedback focused on history-taking, comprehensive management and organisational efficiency. There was less emphasis on negotiation skills and comprehensive assessment (Figure 3). The average feedback by FPs in the mini-CEX forms (16.3 words) was longer than the learning plans (13.7 words). There was more emphasis on specifying ‘areas of improvement’ (per the overall quality evaluation) compared with the learning plans (Figure 1).

## Discussion

This is one of the first sub-Saharan studies evaluating feedback in registrars’ learning plans and mini-CEX forms as evidence of educational and clinical supervision. The general quantity and quality of the feedback across both forms were inadequate, which has implications for the registrars’ ability to engage in self-directed learning.

The feedback in the learning plans lacked elements of effectiveness such as context specificity, being tailored to the trainee’s needs and documented action plans. Other deficiencies were failure to focus on areas needing improvement, such as gaps in clinical knowledge, skills and professional behaviour and not specifying how the supervisor would assist the registrar in addressing these gaps. These findings were similar to previous studies where supervisors struggled to point out registrars’ weaknesses or offer suggestions for improvement or action plans.^29^ Given feedback’s vital role in promoting self-directed learning,^18^ the lack of focus on registrars’ learning needs deprives them of opportunities to reflect on how they could improve their performance. As found previously, the written narrative feedback also minimally reflected registrar responses to feedback.^9,18^

Several studies evaluating mini-CEX forms in HIC^23,39^ found that feedback focused more on adequately and less on inadequately performed areas and did not always include documented action plans. The findings of this study were similar, with a tendency for feedback to focus on adequate performance, highlighting strengths, and neglecting areas that needed improvement, with few action plans included. In addition, written feedback was lacking in many of the forms. Mini-CEX forms have descriptors that guide structured feedback on observed performance,^8^ but these were not used effectively. Possible reasons for supervisors not providing effective feedback could be their multiple roles and responsibilities^33^ and the lack of protected time for educational activities,^40^ especially for direct observations or their lack of skills or training.^5,17,39^ The supervisors in this study often appeared reluctant to provide honest and negative feedback and grade trainee performance when it is poor, as has been seen in previous studies.^1,5^ Other possible reasons for the poor supervisory feedback reported in this study could be deficiencies in trainers’ knowledge and clinical competence, facilitation or interpersonal skills, and awareness of trainee’s learning needs – all factors that have been described in other studies.^2,33^ Organisational factors such as work demands, poor recognition for teaching activities and a lack of a conducive environment could also negatively affect feedback provision.^1^ Some FP supervisors participated in training courses conducted by the academy or the university but ongoing training opportunities could capacitate them better than once off attending training.^41^

In the mini-CEX forms, instead of using competency descriptors, supervisors mainly provided global feedback or non-specific judgements such as ‘satisfactory’, which are vague and do not offer any educational value, as reported previously.^22,23,42^ The inadequate space for written feedback on each competency resulted in supervisors recording feedback anywhere on the forms. The WPBA forms can be modified to so-called ‘supervised learning events’, as done in the United Kingdom,^43^ in which text boxes can replace rating scales with tick boxes to provide narrative feedback. Modified mini-CEX forms with separate spaces to record areas performed well, those that need improvement, and action plans improve educational impact because they act as reminders for supervisors.^36,44^ Although general descriptors offer a rough guide for providing feedback, modifying the forms to include the categories ‘competent’, ‘not competent’, and ‘good’ can guide supervisors to provide more specific feedback.^45^

Most FP supervisors scored highly on all mini-CEX competencies and total and global scores across all years of training. The lenient scoring was evident from the first year of training and did not exhibit the regular progression as seen in previous studies.^45,46^ The global and total scores progression was evident only between the first and third years, in contrast to previous studies demonstrating score progression across all 3 years.^46^ Ideally, feedback should be provided in the areas where registrars scored low. Insufficient supervisor feedback on the areas that scored low could lead to the registrar’s self-assessing by over- or under-estimating their performance against expected standards.^8^ Feedback should improve and be more holistic during the later years compared with the first year, where it could be limited to fewer competencies and be more specific. This avoids overloading junior registrars with feedback, allowing them to apply it in similar situations and enhancing transferability.^6^ More than half of the mini-CEX forms were not signed by FPs or registrars, suggesting a lack of accountability from supervisors and registrars.

At the end of each clinical rotation, FPs and not non-FPs are expected to assess and provide feedback on whether registrars met the initial learning needs identified in their learning plans. Feedback provided by specialists and medical officers who functioned as educational supervisors may not have been aligned with training expectations. This could have been because of non-FP supervisors being unclear about the registrar’s initially identified expected learning outcomes. A previous SA study also reported that registrars received more feedback from non-FP supervisors, which they found useful.^47^ The role of non-FP supervisors in registrar supervision needs further research.

The reliability of WPBAs increases with multiple assessments conducted at various times by different assessors.^22,45^ In this study, registrars had multiple assessors in some districts, but, in others, the mini-CEX forms were completed only by their immediate supervisor. Clinicians functioning as supervisors in clinical settings are neither always trained in medical education^8^ nor equipped to give meaningful feedback.^5,6^ This could negatively impact registrar training, even more so in districts where only one supervisor provides feedback. Regular faculty development is needed to understand the educational value of feedback.^22,27^ Identifying a core group of well-trained supervisors to provide feedback when conducting WPBAs^5^ could be another strategy to address the reliability of mini-CEX assessments.^27^

Feedback in the learning plans focused on ethics, professionalism, clinical knowledge and skills and learning behaviours. This was in contrast to a study where professionalism was the learning goal least identified by trainees.^48^ Feedback provided by specialists and medical officers was much more comprehensive, covered many competencies and was aligned to registrars’ learning needs (Figure 2). Supervisors’ ability to provide feedback on appropriate history-taking and physical examination skills is critical in workplace learning, as clinical diagnosis depends on these registrar skills.^5^ Mini-CEX feedback mostly covered history-taking, physical examination, comprehensive management and organisational efficiency. Clinical reasoning was the least covered. This differed from previous studies where feedback focused least on professionalism and organisational efficiency.^49^ As reported previously, there was no alignment of many feedback areas to the registrars’ learning needs.^49^ Family physicians provided scanty feedback on soft skills, such as clinical reasoning or patient negotiation skills (Figure 3). High scores given by FP supervisors on all competencies suggested insufficient skills to evaluate registrars critically, especially for soft skills. Ideally, more narrative feedback should be offered on identified learning needs where supervisors gave low scores.

This is the first sub-Saharan study to explore the significance of training sites and supervisor characteristics on the quality of feedback. While one district (D3) had fewer FPs involved in training, registrars received more comprehensive feedback than other districts with more FPs. This infers that feedback does not depend on the number of supervisors but is context-specific, dependent on willingness and effort, understanding of feedback characteristics^5^ and assessment literacy.^7,22^ All supervisors and registrars involved in decentralised clinical training require ongoing faculty development training on what constitutes adequate feedback to maximise this essential element of workplace-based training and assessments in decentralised settings. There are several training options that could supplement the existing training. One option is to conduct practical sessions using videos of actual registrar consultations and the supervisory feedback that could be critiqued by pairs or groups of FPs as done in other settings.^50^ Another strategy could capitalise on the peer coaching or co-teaching^1^ opportunities provided by observed consultations – pairs of junior and senior FPs should provide feedback to registrars after these sessions. Similarly, feedback sessions between FPs and registrars in combined sessions with medical students or interns at clinics or hospitals provide opportunities for registrars to practise and internalise effective feedback. Finally, and most importantly, FPs should promote an empathic learning culture based on constructive feedback, even at the lowest family medicine training level, at the primary healthcare facilities.

### Limitations

Only written feedback was evaluated; verbal feedback during direct observations was not included. Scores and feedback were compared across the different sets of registrars in the three years but not for the same registrar across training years. Although it is difficult to make high-quality assessments in postgraduate training because of low numbers,^27^ all registrars’ portfolios were included to maximise participation. The principal author constantly engaged with co-authors on coding and analysis, improving internal and external validity. The study was conducted in the decentralised sites of one university, which could affect the generalisability of the results.

### Recommendations

Given the integral role of feedback in effective clinical and educational supervision, we suggest there is a need to improve the feedback supervisors provide to trainees and how trainees utilise the feedback. Supervisors and registrars need regular faculty development on various aspects of effective feedback. Training for supervisors should focus on how to give specific feedback according to registrar performance, especially on softer skills such as clinical reasoning and professionalism, along with other hard skills such as history-taking and physical examination, how to develop action plans and assist in implementing the action plans. For the registrars, the training should focus on utilising the written feedback to develop as self-directed lifelong learners.

## Conclusion

This study evaluated supervisors’ feedback in family medicine registrars’ LPs. Supervisor feedback was shown to be inadequate, often very general and not very helpful in developing action plans to improve registrars’ skills. The lack of detail tailored to individual needs could negatively impact the training of health workers. Family physicians’ supervisory role is vital to ensure the training of skilled family physicians to strengthen primary healthcare and district health systems, which should translate to better health outcomes in communities. Future research on feedback in postgraduate family medicine training programmes in similar contexts could help to improve the quality of this cadre of health professionals.
